# Rotational stability and clinical outcomes of a new one piece toric intraocular lens with anchor-wing haptics

**DOI:** 10.1186/s12886-021-02240-7

**Published:** 2022-01-15

**Authors:** Iichiro Sugita, Tomoichiro Ogawa, Kazuo Ichikawa, Takahide Okita, Kazuno Negishi, Tadashi Nakano, Hiroshi Tsuneoka

**Affiliations:** 1 Sugita Eye Hospital, 5-1-30, Sakae, Naka-ku, Nagoya-shi, Aichi 460-0008 Japan; 2 Department of Ophthalmology, the Jikei University School of Medicine, 3-25-8, Nishi-Shimbashi, Minato-ku, Tokyo, 105-8461 Japan; 3 Miyamaedaira Ogawa Eye Clinic, 19-14, Nanpeidai, Miyamae-ku, Kawasaki-shi, Kanagawa 216-0024 Japan; 4 Chukyo Eye Clinic, 12-22, Sanbonmatsu-cho, Atsuta-ku, Nagoya-shi, Aichi 456-0032 Japan; 5 Department of Ophthalmology, Keio University School of Medicine, 35 Shinanomachi, Shinjuku-ku, Tokyo, 160-8582 Japan

**Keywords:** Toric intraocular lens, Intraocular lens, Corneal astigmatism, Cataract, Cataract surgery, Rotational stability, Anchor-wing haptics

## Abstract

**Background:**

To evaluate the safety and efficacy of a new toric intraocular lens (IOL) with anchor-wing haptics.

**Methods:**

The new toric IOL with anchor-wing haptics (NS60YT, NIDEK Co., Ltd.) was implanted in eligible patients with age-related cataracts with preoperative corneal astigmatism of 1.0 D or greater at a university hospital and two private hospitals in Japan. The following IOL cylinder powers were evaluated: 1.50 D (NS60YT3), 2.25 D (NS60YT4), 3.00 D (NS60YT5) and 4.50 D (NS60YT7). All patients were assessed out to 12 months postoperatively. The primary endpoint was visual acuity (VA) with spherical addition at 6 months postoperatively, and the primary analysis calculated the proportion of eyes with VA with spherical addition of 0.1 logMAR or better. The magnitude of rotation was compared to the intended axis of IOL implantation at each postoperative examination. Adverse events were evaluated for the safety analysis.

**Results:**

This study enrolled 64 eyes of 53 patients. At 6 months postoperatively, for all IOL powers, VA with spherical addition of 0.1 logMAR or better was achieved in 90% [95% confidence interval (CI): 80–96] of eyes. The mean IOL rotation was 5.3 ± 4.3° at 12 months postoperatively. The mean magnitude of rotation ranged from 1.9° to 2.5° between each postoperative examination from 1 day to 12 months. There were no vision-threatening intraoperative or postoperative complications for the duration of the study.

**Conclusions:**

The NS60YT IOL remained stable after implantation and was efficacious for treating 1.00 D or greater astigmatism in patients with senile cataracts.

**Trial registration:**

This study was registered at ClinicalTrials.gov (NCT03242486) on August 8, 2017 - Retrospectively registered.

## Background

The reduction of astigmatism during cataract surgery is an important factor in visual function and postoperative patient satisfaction. Approximately 35% of patients with cataract have preoperative corneal astigmatism of 1.0 D or greater and approximately 20% have 1.5 D or greater astigmatism [1–4]. Residual astigmatism after phacoemulsification and intraocular lens (IOL) implantation is a significant cause of suboptimal vision, increased spectacle dependence or patient dissatisfaction [5, 6]. There are a variety of methods currently used for treating cataracts with co-existing corneal astigmatism, including but not limited to, implantation of supplementary sulcus-fixated IOLs, implantation of pinhole IOLs, laser refractive surgery for a corneal “touch-up”, or toric IOL. The advantages of toric IOL implantation include a wider range of correction and the use of one procedure to simultaneously correct the refractive error, reducing the burden on the patient and the surgeon [7]. The Aktis toric (Model NS60YT; Nidek Co., Ltd.) is a newly developed IOL that is based on the existing monofocal Nex-Acri® AA 1P IOL platform (Nidek Co., Ltd.). Rotational stability for this IOL is maintained by anchor-wing haptics. In this prospective, multicenter study, we present the stability and clinical outcomes of NS60YT implantation for the correction of moderate to high astigmatism.

## Methods

This single-arm, open-label, multicenter, prospective clinical study evaluated implantation of the NS60YT in patients with age-related cataracts at a university hospital and two private hospitals in Japan. This study was approved by the Jikei University Hospital Institutional Review Board for Medical Devices, the Sugita Eye Hospital Institutional Review Board, and the Joint Institutional Review Board of the hospitals. The study adhered to the Declaration of Helsinki and the Japanese Ministerial Ordinance on Good Clinical Practice for Medical Devices (GCP). This manuscript adheres to the CONSORT guidelines. The study protocol is available at https://clinicaltrials.gov. Written informed consent was obtained from all patients before participation. The study period was from December 2014 to November 2017. The duration of follow up was 12 months postoperatively.

### Intraocular lens

**Fig. 1:**
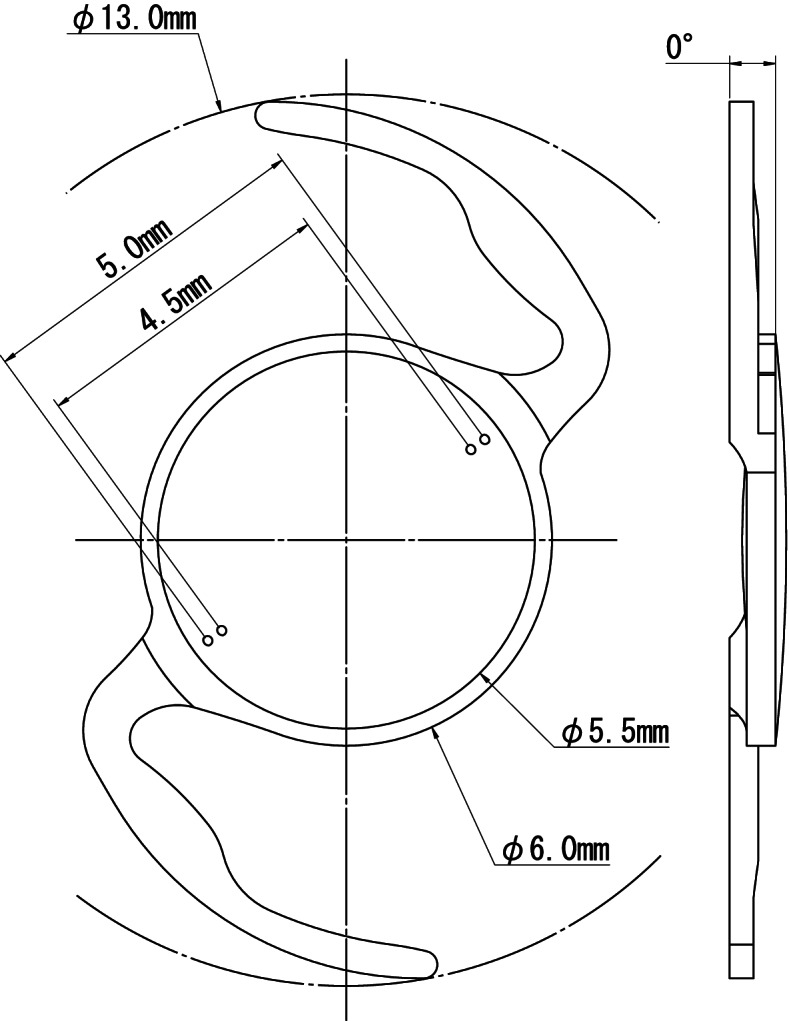
Design specifications of the NS60YT intraocular lens

The NS60YT is a single-piece toric IOL based on the Nex-Acri® series (Nidek Co., Ltd.) (Fig. 1). The optical diameter is 6.0 mm and the overall diameter is 13.0 mm including the anchor-wing haptics. The lens is a biconvex lens and the rear surface is aspheric (− 0.15 μm). The cylindrical power is placed on the front of the lens for the correction of corneal astigmatism. The IOL consists of hydrophobic soft acrylic resin with an ultraviolet absorber and proprietary material to enhance compatibility with the injector. The lens has two dots on either side of the optic periphery, indicating the flattest meridian for marking the cylinder axis. This study evaluated 4 different toric IOL powers as follows: 1.50 D, 2.25 D, 3.00 D and 4.50 D (Table 1).

**Table 1:** Cylindrical power of each model of the NS60YT intraocular lens

Model name	IOL surface power (D)	Power at the Corneal Plane (D)
NS60YT3	1.50	1.05
NS60YT4	2.25	1.57
NS60YT5	3.00	2.08
NS60YT7	4.50	3.11

### Patients

Patients were included if they were over 40 years old with age-related cataracts in one or both eyes with a pupil diameter of 5.0 mm or larger at mydriasis and had 1.0 D or greater preoperative corneal astigmatism. Other inclusion criteria were, a predicted postoperative astigmatism less than 0.5 D, and the predicted postoperative visual acuity (VA) with spherical addition of 0.1 logMAR or better. “VA with spherical addition” is visual acuity measured under conditions where a spherical trial lens is added to correct the postoperative spherical refractive error determined before surgery for each patient.

Patients were excluded if they had irregular corneal astigmatism, axial length > 28 mm, and other diseases or complications that might affect the efficacy and safety of IOL implantation.

### Preoperative examination

Within 60 days prior to surgery, the patient underwent thorough ophthalmic examinations including, slit lamp microscopy, corneal topography (OPD-Scan® III; Nidek Co., Ltd.) for measurement of corneal astigmatism, measurement of pupil diameter, tonometry, fundus examination, and axial length measurement (IOLMaster®; Carl Zeiss Meditec AG).

### Calculation of intraocular lens power using a toric calculator

The IOL spherical power was selected by the surgeon based on the desired postoperative refractive error for each patient using the biometry values. The SRK/T formula was used for all eyes. The A-constants (119.7) were adjusted for each clinical site based on the constant for the Nex-Acri® AA 1P that is the same shape as NS60YT. The Nidek Toric Calculator For Clinical Trials was used to select cylinder power and to calculate the angle of implantation. Data entry of the preoperative corneal astigmatism, the surgically induced astigmatism for each surgeon, and the location of the incision allows calculation of the predicted postoperative corneal astigmatism, the IOL implantation axis, and the predicted postoperative residual astigmatism for each model. Based on these variables, the surgeons selected the optimum IOL model that predicted the lowest postoperative astigmatism.

### Surgery

Once the axis of implantation was verified with topographic data just prior to surgery, the patient was seated at a slit lamp and the surgeon used a gentian violet pen to mark the 3-o’clock, 6-o’clock and 9-o’clock positions. Intraoperatively, once the patient is supine and just prior to IOL implantation, the absence of head tilt is verified, and the gentian violet marks are used with a protracting device to find the target meridian. The target meridian was marked on the corneo-scleral junction. A paracentesis was created and a dispersive viscoelastic was delivered to the anterior chamber to protect the corneal endothelium. A 2.2 to 2.5 mm corneal, scleral or corneo-scleral incision was created. A well-centered manual capsulorhexsis was created and the cataractous lens was removed by phacoemulsification and aspiration. A cohesive viscoelastic was delivered followed by delivery of the IOL into the capsular bag using an injector (Nex-IJ; Nidek Co., Ltd.). The IOL was unfolded and rotated close to the target meridian and the viscoelastic was removed. The IOL was rotated to ensure the toric axis marks aligned with the target meridian and the lens was centered. Although an automated system for IOL alignment would have be ideal, the study protocol was written in 2013 and we did not have equipment or resources to acquire automated intraoperative images at that time. In patients scheduled for bilateral surgery, the fellow eye underwent surgery after the initial eye was evaluated at 1 week postoperatively and there were no complications.

### Postoperative examination

Postoperative examinations were performed the day after surgery (1–2 days), 1 week after surgery (7–14 days), 1 month after surgery (30–60 days), 3 months after surgery (90–150 days), 6 months after surgery (180–240 days), and 12 months after surgery (360–420 days). Postoperative examinations included slit lamp microscopy, tonometry, fundus examination, measurements of corneal curvature with corneal topography, UCVA, VA with spherical addition (VA corrected with “intended postoperative spherical refractive error”), sphere-corrected visual acuity (VA corrected with “actual postoperative spherical refractive error”), best corrected distance visual acuity (BCVA), IOL axis evaluation, and a patient questionnaire (only at 6 months postoperatively). Sphere-corrected VA is the visual acuity when only the sphere was corrected from the VA with spherical addition based on the postoperative spherical error.

The IOL axis was measured manually using slit lamp photography or from images obtained with anterior segment imaging devices (KATS-1000; Konan medical Co., Ltd. and Casia; Tomey Corp.). The patient was seated at the slit lamp and the correct head position (no tilt) was verified prior to acquiring photographs. Deviation between the corneal astigmatic axis and the axis marks on the IOL was determined by using a protractor. The image was regarded as horizontal, and the angle was measured between the horizontal line and the toric mark. The patient questionnaire classified current vision into five rankings as follows: “very satisfied”, “satisfied”, “neither satisfied nor dissatisfied”, “dissatisfied”, and “very dissatisfied”.

Data were collected on all postoperative adverse events irrespective of causality. The following adverse events that may occur after cataract surgery were summarized as anticipated adverse events at every postoperative examination: secondary cataract (requiring posterior capsulotomy), macular degeneration, macular edema, hypopyon, infectious and noninfectious endophthalmitis, pupil block, retinal detachment, corneal edema, iritis, increased intraocular pressure (IOP), and IOL dislocation. Increased IOP was defined as 23 mmHg or higher and an increase of 5 mmHg or higher compared to the preoperative IOP.

### Statistical analysis

We used previous studies of toric IOLs that targetted emmetropia as the historical control to determine the number of cases that achieved a postoperative UCVA of 0.1 logMAR or better [8–10]. The analysis indicated 47 eyes were required for NS60YT3, NS60YT4, NS60YT5 (significance level α = 0.05, power 1-β = 0.80), based on the threshold response rate (π0) 0.5 and the expected response rate (π1) 0.7. For high astigmatism (NS60YT7), data were collected on only 5 eyes due to the lack of appropriate patients.

The primary endpoint was the VA with spherical addition 6 months after surgery, and the primary analysis calculated the proportion of VA with spherical addition of 0.1 logMAR or better and a 95% confidence interval (CI). The Clopper-Pearson method was used to calculate 95% CIs. Secondary endpoints included UCVA, sphere-corrected VA, BCVA, refractive cylinder correction, and the magnitude of IOL rotation. Visual acuity was recorded in decimal notation at all sites, however for statistical analysis and consistency, all values were reported in logMAR. The magnitude of IOL rotation was calculated as the absolute value of the difference between the actual IOL axis and preoperative IOL insertion axis calculated with the Nidek Toric Calculator For Clinical Trials.

Safety endpoints included the presence, absence, and incidence of adverse events after IOL insertion, and the presence or absence of anticipated adverse events. SAS (Version 9.3, SAS Institute Inc.) was used for statistical analysis. *P* < 0.05 was considered statistically significant.

## Results

The study sample was comprised of 64 eyes of 53 patients. Two eyes of one patient were excluded from all analyses due to non-compliance with GCP because the consent forms were misplaced. One patient died after 6 months examination due to reasons unrelated to cataract surgery. Therefore, the full analysis set for efficacy, included 62 eyes of 52 patients at 6 months postoperatively and 61 eyes of 51 patients at 12 months postoperatively. Table 2 presents patient demographics, baseline examination data and IOL calculation data. The mean age at enrollment was 68.6 ± 9.5 years (Table 2). The mean targeted postoperative refraction was − 1.1 ± 1.2 D (Table 2).

**Table 2:** Demographics and preoperative data of patients scheduled to undergo cataract surgery with implantation of the NS60YT intraocular lens

Parameter	All models	NS60YT3	NS60YT4	NS60YT5	NS60YT7
Number of eyes, n	62	21	20	15	6
Number of patients, n	52	20	20	13	6
Age					
Mean (years) ± SD	68.6 ± 9.5	69.1 ± 9.2	68.1 ± 10.2	66.7 ± 10.7	73.2 ± 4.6
Minimum value, maximum value	42, 84	54, 84	48, 84	42, 79	66, 79
Sex, n					
Male	27	8	8	9	2
Female	35	13	12	6	4
Mean pupil diameter (mm) ± SD	7.43 ± 0.82	7.57 ± 0.62	7.25 ± 0.71	7.32 ± 0.99	7.75 ± 1.25
Mean AL (mm) ± SD	24.21 ± 1.33	24.01 ± 1.35	23.99 ± 1.47	24.77 ± 1.21	24.24 ± 0.92
Mean preoperative corneal astigmatism (D) ± SD	1.73 ± 0.54	1.32 ± 0.18	1.63 ± 0.24	1.95 ± 0.30	2.99 ± 0.47
Preoperative corneal astigmatic axis, n (%)					
WTR astigmatism (0° to 29°, 150° to 180°)	29 (47)	7 (33)	9 (45)	10 (67)	3 (50)
OBL astigmatism (30° to 59°, 120° to 149°)	1 (2)	0 (0)	1 (5)	0 (0)	0 (0)
ATR astigmatism (60° to 119°)	32 (52)	14 (67)	10 (50)	5 (33)	3 (50)
Mean postoperative target spherical refraction(D) ± SD	−1.1 ± 1.2	−0.8 ± 1.3	− 0.9 ± 1.0	−1.6 ± 1.4	−1.3 ± 1.3

*ATR* Against-the-rule, *AL* Axial length, *IOL* Intraocular lens, *OBL* Oblique, *SD* Standard deviation, *WTR* With-the-rule

### Primary endpoint - visual acuity with spherical addition

Table 3 presents the postoperative VA with spherical addition of 0.1 logMAR or better and the 95% CI. At 6 months, the primary endpoint was achieved in 90% (56/62 eyes) for all models, 100% (21/21 eyes), 80% (16/20 eyes), 93% (14/15 eyes), and 83% (5/6 eyes) for NS60YT3, NS60YT4, NS60YT5 and NS60YT7, respectively. At 12 months, 87% achieved the primary endpoint for all models.

**Table 3:** Visual acuity with spherical addition of 0.1 logMAR or better, 6 and 12 months after cataract surgery with implantation of the NS60YT intraocular lens

Postoperative		All models	NS60YT3	NS60YT4	NS60YT5	NS60YT7
6 months	Number of eyes, n	62	21	20	15	6
	VA with spherical addition of 0.1 logMAR or better, n (%)	56 (90)	21 (100)	16 (80)	14 (93)	5 (83)
	95% CI (%)	80, 96	84, 100	56, 94	68, 100	36, 100
12 months	Number of eyes, n	61	21	20	14	6
	VA with spherical addition of 0.1 logMAR or better, n (%)	53 (87)	18 (86)	18 (90)	12 (86)	5 (83)
	95% CI (%)	76, 94	64, 97	68, 99	57, 98	36, 100

*CI* Confidence interval, *UCVA* Uncorrected visual acuity

### Other efficacy parameters and safety parameters

#### Visual acuity

Table 4 presents the UCVA, VA with spherical addition, sphere-corrected VA, BCVA and subjective cylindrical power up to 12 months postoperatively. VA with spherical addition, sphere-corrected VA and BCVA remained stable until 12 months postoperatively after 1 month postoperatively (Table 4). At 12 months postoperatively, the residual subjective cylindrical power (the dioptric power determined during subjective refraction) was less than 0.50 D in 59% (36/61 eyes) and less than 1.00 D in 89% (54/61 eyes) for all models combined.

**Table 4:** Mean efficacy parameters for eyes that underwent cataract surgery with NS60YT intraocular lens implantation

	1 day	1 week	1 month	3 months	6 months	12 months
UCVA (logMAR)	0.29 ± 0.38	0.28 ± 0.40	0.27 ± 0.42	0.28 ± 0.43	0.29 ± 0.45	0.28 ± 0.42
VA with spherical addition (logMAR)	0.08 ± 0.19	0.04 ± 0.17	0.03 ± 0.19	0.03 ± 0.16	0.02 ± 0.17	0.00 ± 0.11
Sphere-corrected VA (logMAR)	− 0.01 ± 0.11	− 0.04 ± 0.09	− 0.05 ± 0.07	−0.05 ± 0.07	−0.05 ± 0.07	−0.05 ± 0.07
BCVA (logMAR)	− 0.05 ± 0.09	−0.08 ± 0.07	−0.09 ± 0.05	−0.10 ± 0.05	−0.10 ± 0.06	−0.10 ± 0.06
Subjective cylindrical power (D)	0.54 ± 0.61	0.53 ± 0.56	0.55 ± 0.46	0.61 ± 0.45	0.58 ± 0.49	0.55 ± 0.47

*BCVA* Best corrected visual acuity, *IOL* Intraocular lens, *logMAR* Logarithm of the minimum angle of resolution, *UCVA* Uncorrected visual acuity, *VA* Visual acuity

**Fig. 2:**
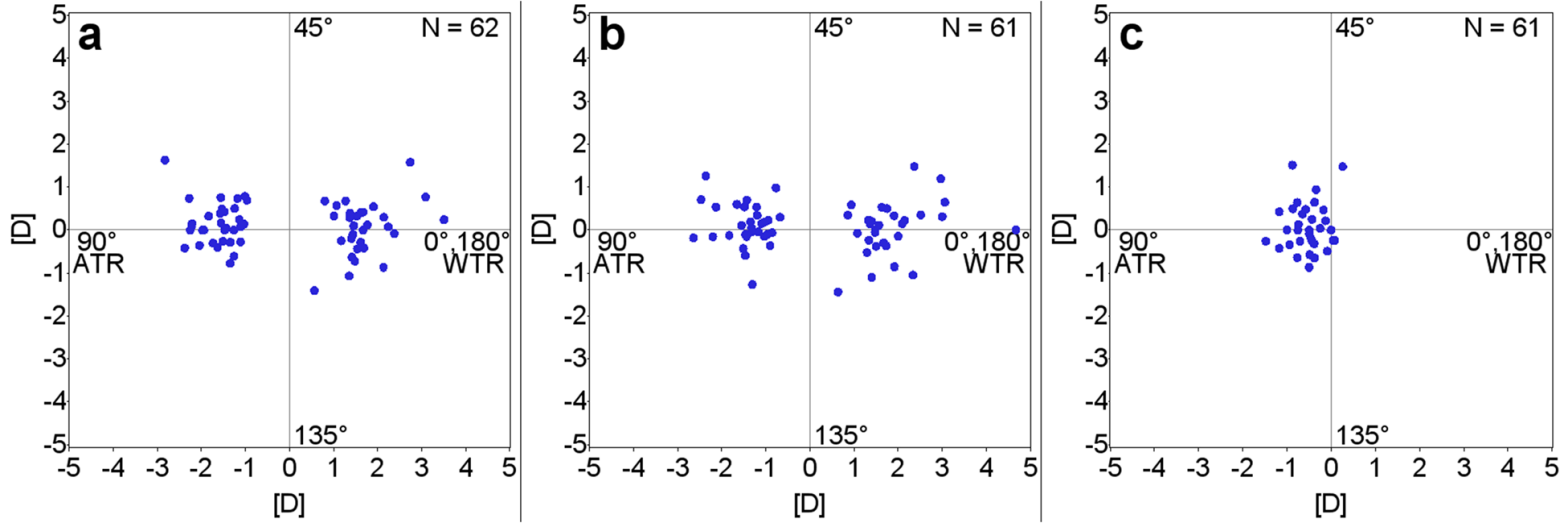
Corneal astigmatism and postoperative subjective cylinder components (ATR = against-the-rule; WTR = with-the-rule). **a** Preoperative corneal astigmatism. **b** 12 months postoperative corneal astigmatism. **c** 12 months postoperative subjective cylinder components

Figure 2 presents the double angle polar plots. There were no remarkable changes observed between the preoperative corneal astigmatism (Fig. 2a) and the 12 months postoperative corneal astigmatism (Fig. 2b). However, in eyes with subjective cylinder at 12 months postoperatively, the cylinder components tended towards against-the-rule (ATR) astigmatism (Fig. 2c).

#### Intraocular lens rotation (including additional analysis)

**Fig. 3:**
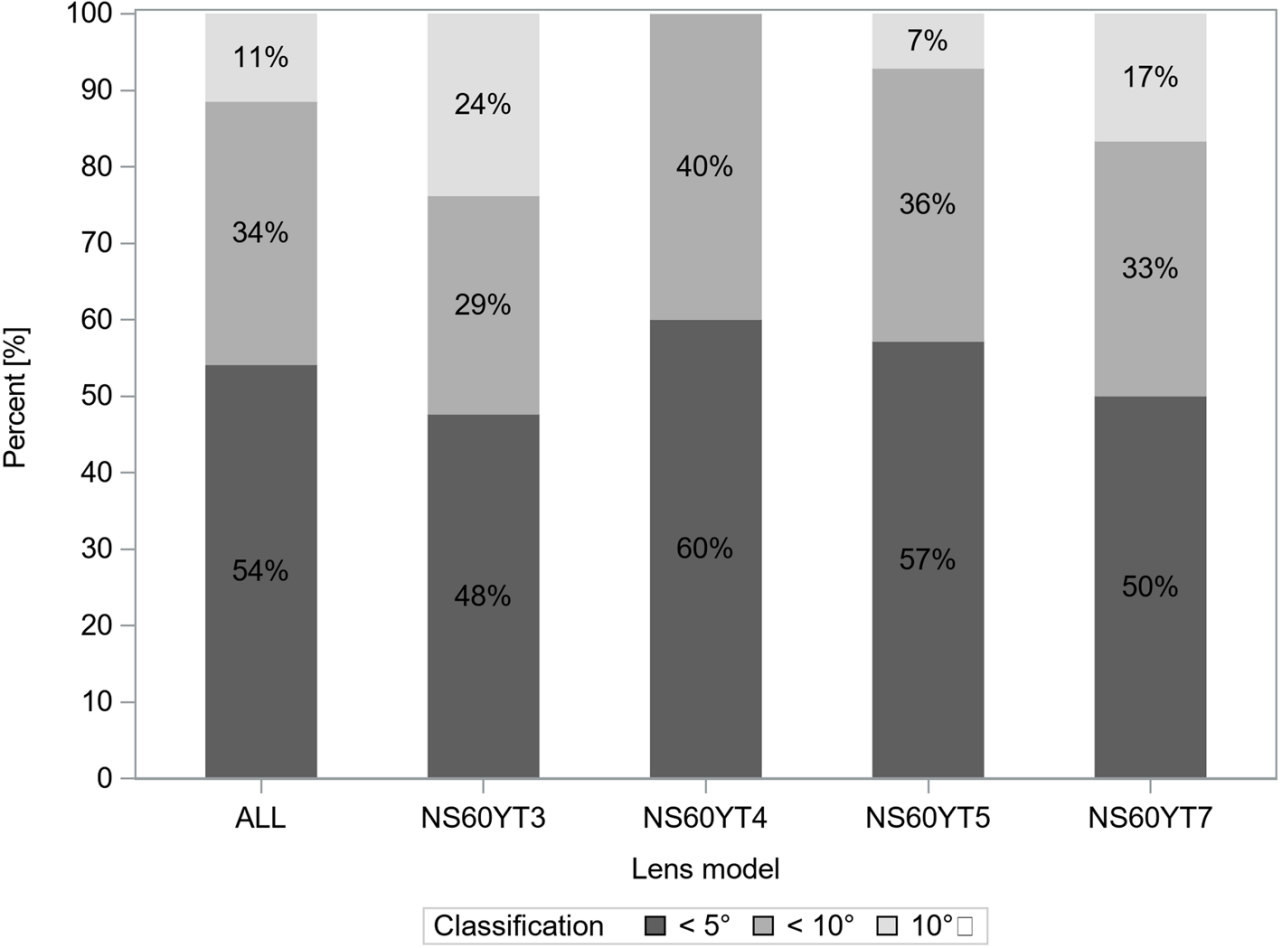
Distribution of intraocular lens rotation at 12 months after surgery

Figure 3 presents the distribution of the rotation (absolute value) at 12 months compared to the axis of IOL insertion. A deviation less than 10° between the IOL axis and the insertion axis was noted in 89% (54/61 eyes) of all models. No cases required repositioning surgery due to postoperative IOL rotation.

Additional analysis indicated that the magnitude of rotation the day after surgery was 5.5°, and the mean rotation between each examination after day 1 was 2.5° or lower (Table 5). A rotation of 5.0° or more occurred in 52% by day 1, however, a rotation of 5.0° or more after day 1 was 7 to 13% for each period (Table 5). At 12 months postoperatively, the IOL rotated clockwise in 61% (37/61) of eyes, counterclockwise in 31% (19/61) of eyes, and the IOL did not rotate in 8% (5/61) of eyes. The direction of rotation and the preoperative corneal cylinder axis were not statistically associated (Fisher’s exact test, *P* = 0.781).

**Table 5:** Rotational stability of the NS60YT intraocular lens

Postoperative	Number of eyes, n	Rotation amount (absolute value) ± SD(°)	Lens rotation amount, n (%)		
			Less than 5.0°	5.0° or more and less than 10.0°	10.0° or more
1 day	61	5.5 ± 3.8	29 (48)	22 (36)	10 (16)
1 day to 1 week	61	2.5 ± 2.7	55 (90)	4 (7)	2 (3)
1 week to 1 month	61	2.2 ± 1.7	55 (90)	6 (10)	0 (0)
1 month to 3 months	61	2.0 ± 1.8	56 (92)	5 (8)	0 (0)
3 months to 6 months	61	2.1 ± 2.0	53 (87)	8 (13)	0 (0)
6 months to 12 months	61	1.9 ± 1.5	57 (93)	4 (7)	0 (0)
1 day to 12 months	61	3.1 ± 2.9	47 (77)	11 (18)	3 (5)

*SD* Standard deviation

#### Patient satisfaction

A questionnaire at 6 months queried patient satisfaction. For all models, the results were “very satisfied” 52% (32/62 eyes), “satisfied” 40% (25/62 eyes), “neither satisfied nor dissatisfied” 3% (2/62 eyes), “dissatisfied” 5% (3/62 eyes), and “very dissatisfied” 0% (0/62 eyes). Thus, “very satisfied” and “satisfied” accounted for 92% of eyes.

#### Safety

Postoperative adverse events where a causal relationship could not be ruled out included 4 eyes (6%) with mild posterior capsule opacification. Anticipated adverse events (defined separately at the beginning of the study) developed in 6 eyes of 5 patients. These included 2 eyes (3%) with secondary cataract (requiring posterior capsulotomy) and 4 eyes (6%) with increased IOP. One patient died during the course of the study due to multiple organ failure which was a non-ocular adverse event and had no causal relationship to cataract surgery or IOL implantation.

## Discussion

This prospective evaluation of a new toric IOL (NS60YT) indicated the majority of eyes had excellent vision after cataract surgery. The primary endpoint of the study was VA with spherical addition for this Japanese population. This index was used as the primary endpoint taking into consideration the tendency of lens selection in Japan. Japanese cataract patients tend to prefer some nearsightedness instead of emmetropia postoperatively. Using an VA with spherical addition as the primary endpoint enabled comparison with the published literature on toric IOLs targeting emmetropia despite targeting postoperative myopia (in the current study).

At 6 months postoperatively, 90% (56/62 eyes) of all eyes achieved VA with spherical addition of 0.1 logMAR or better. The lower limit (80%) of the 95% CI exceeded the expected response rate of 70%, which was the basis for determining the number of cases. This outcome indicates, that patients achieved equivalent or better postoperative visual acuity with the NS60YT in comparison with commercially available toric IOLs as the historical controls.

In contrast to the VA with spherical addition, sphere-corrected VA is the visual acuity obtained by further correcting the postoperative spherical refractive error only. In addition, the BCVA is the best visual acuity obtained by further correcting the residual postoperative cylindrical power from the sphere-corrected VA. This means that the difference between the VA with spherical addition and the sphere-corrected VA is the spherical refractive error with respect to the target refractive power. Additionally, the difference between the sphere-corrected VA and the BCVA is the cylindrical refractive error. Twelve months after surgery, the outcomes of the current study indicate a sphere-corrected VA of less than 0.1 logMAR in only one eye (2%), and 0.1 logMAR or better in the other 60 eyes (98%). This outcome indicates excellent cylinder correction with the NS60YT.

Vector analysis indicated that the postoperative subjective cylinder had a distribution toward ATR astigmatism. This trend is similar to the outcomes of wavefront analysis reported by Ninomiya et al. [11]. The primary cause of ATR astigmatism after surgery could be due to the effect of posterior corneal astigmatism. As this study was designed in 2014, the protocol did not incorporate the effect of posterior corneal curvature. The current study used only the anterior corneal surface to evaluate corneal astigmatism, and the Nidek Toric Calculator For Clinical Trials did not consider the posterior corneal astigmatism for lens selection. Koch et al. [12] have reported that posterior corneal astigmatism causes overcorrection of with-the-rule (WTR) astigmatism and decreases the correction of ATR astigmatism when the lens selection is based on the anterior corneal astigmatism only. Hence, our outcomes are consistent with Koch et al’s observations [13]. Savini et al. have published nomograms for selecting the IOL power when only the anterior corneal astigmatism is used. For preoperative WTR astigmatism, residual astigmatism is calculated based on the result from subtracting 0.59 D to 0.70 D from the predicted postoperative corneal astigmatism [14]. For preoperative ATR astigmatism, residual astigmatism is calculated based on the result of adding 0.32 D to 0.70 D to the predicted postoperative corneal astigmatism [14]. The current version of the Nidek Toric Calculator includes the Baylor nomogram to compensate for the posterior surface power when only anterior corneal surface data are used [12, 13].

Rotational stability of a toric IOL is crucial for achieving accurate cylinder correction. Every 1° rotation of a toric IOL from the intended axis can decrease cylinder correction efficacy by 3.3%. For example, a postoperative toric IOL rotation over 30° results in additional astigmatism [15]. The outcomes of the current study indicate the NS60YT has good rotational stability. For example, the mean IOL rotation at 6 months was 5.0 ± 4.4°for all models combined which is well within the range reported for other IOLs. Previous studies have reported rotation of single-piece toric IOLs between the intended axis and the observed axis from 2° to 9° at 6 months postoperatively [9, 16–20]. Additionally, in the current study, the magnitude of rotation ranged from 1.9° to 2.5° between each postoperative examination from 1 day to 12 months and the mean rotation was 3.1 ± 2.9°. The tendency to rotate early in the postoperative period and then stabilize was similar to the previous literature on existing single-piece toric IOLs [21–23].

The anchor wing-haptic design contributes to the stability of the NS60YT toric IOL. The 90° orientation of the anchor wing-haptic design makes it difficult to generate a force in the rotational direction from compression due to the pressure from capsular contraction. Additionally, the width between the haptic shoulders is based on an average capsular diameter. The design geometry allows a secure fit within the capsular bag using the shoulders to stabilize the IOL without relying on the overall haptics (‘arms’) of the IOL.

Table 6 presents data on seven cases with a rotation of 10° or greater at 12 months postoperatively relative to the IOL insertion axis. Five cases (except Cases 3 and 4) rotated 10° or more at 1 day. In Case 4, a rotation of 10° or more was observed initially at 1 month. This case had an incomplete closed capsulorhexis with 2 tears. Perhaps postoperative healing and suboptimal capsular contraction due to the tears may have played a role in the rotation. Case 3 presented with a rotation of 10° or more initially at 12 months. The subsequent rotations were as follows: − 4° at 1 week; − 2° at 1 month; − 9° at 3 months; − 9° at 6 months; and − 13° at 12 months. We are at a loss to explain the IOL rotation between visits. However we can rule out abnormal head positioning as experienced technicians, familiar with the equipment ensured the patient’s head was in correct position prior to acquiring images. Although abnormal head position may have been missed at one visit, it would be highly unlikely in consecutive visits with the same patient. Additionally, the observation that the majority of patients were not symptomatic concurs with the low magnitude of rotation reported in the current study and indicates that relatively good image acquisition techniques and accurate measurements were used in the current study.

**Table 6:** Cases with rotation of 10° or greater at 12 months postoperatively

	Case 1	Case 2	Case 3	Case 4	Case 5	Case 6	Case 7
Amount of rotation (°)^a^	15	−16	−13	10	−18	18	−12
Expected IOL axis angle (°)	4	10	95	177	87	94	161
IOL axis angle (°)	19	174	82	7	69	112	149
When the rotation exceeded 10°	1 day	1 day	12 months	1 month	1 day	1 day	1 day
Model	NS60YT7	NS60YT3	NS60YT5	NS60YT3	NS60YT3	NS60YT3	NS60YT3
Spherical power (D)	17.0	19.0	21.5	20.0	21.5	18.0	20.0
AL (mm)	25.50	25.00	23.61	24.85	23.13	25.17	25.28
Age (y)	72	63	45	72	58	59	70
Anterior capsulotomy	CCC	CCC	CCC	CCC with 2-tear	CCC	CCC	CCC
VA with spherical addition (logMAR)	−0.08	0.22	−0.18	0.15	−0.08	−0.08	0.05
Sphere-corrected VA (logMAR)	−0.08	0.22	−0.18	0.05	−0.08	− 0.18	0.00
BCVA (logMAR)	−0.18	0.05	−0.18	0.00	−0.08	−0.18	− 0.08
(Cylinder correction amount)	(−1.75 D)	(−1.00 D)	(−0.50 D)	(− 0.25 D)	(−1.50 D)	(− 0.25 D)	(−0.75 D)
Questionnaire results (at examination of 6 months)	Very satisfied	Satisfied	Satisfied	Very satisfied	Very satisfied	Satisfied	Satisfied

*AL* Axial length, *BCVA* Best corrected visual acuity, *CCC* Continuous curvilinear capsulorhexis, *IOL* Intraocular lens, *UCVA* Uncorrected visual acuity, *VA* Visual acuity

^a^Rotation amount: plus = counterclockwise, minus = clockwise

The cause of rotation in this case is unknown, however, the patient was asymptomatic and there were no postoperative sequelae. In cases with 10°or greater postoperative IOL rotation, we recommended a re-rotation if the patient was symptomatic. In these cases, the VA with spherical addition was 0.1 logMAR or better with Cases 2 and 4 excluded, and the sphere corrected VA was 0.05 logMAR or better with Case 2 excluded. Patient satisfaction with their vision remained high in all cases with a rotation of 10° or more.

This study evaluated the difference of the IOL axis at each postoperative examination relative to the insertion axis. Therefore, the evaluation included assessment of the IOL axis deviation intraoperatively and the rotation from the early postoperative phase onwards. To verify the rotation from the insertion axis more accurately, deviation between the expected insertion axis and the actual insertion axis needs to be evaluated.

## Conclusions

In summary, the outcomes of this study indicate that a new toric IOL with anchor-wing haptics is safe and effective for implantation during cataract surgery. Axis rotation was very low in the majority of cases and clinically insignificant and repositioning of the IOL was not warranted in any patient. Implantation of this toric IOL provides satisfactory postoperative visual acuity and good patient satisfaction.

## Data Availability

The datasets used and analyzed during the current study are available from the corresponding author on reasonable request.

## Abbreviations

ATR: Against-the-rule
AL: Axial length
BCVA: Best corrected visual acuity
CCC: Continuous curvilinear capsulorhexis
CI: Confidence interval
D: Diopter
GCP: Good clinical practice
IOL: Intraocular lens
IOP: Intraocular pressure
LogMAR: Logarithm of the minimum angle of resolution;
OBL: Oblique
SD: Standard deviation
UCVA: Uncorrected visual acuity
VA: Visual acuity
WTR: With-the-rule
